# Vertical ridge augmentation feasibility using unfixed collagen membranes and particulate bone substitutes: A 1‐ to 7‐year retrospective single‐cohort observational study

**DOI:** 10.1111/cid.13084

**Published:** 2022-03-24

**Authors:** Jung‐Seok Lee, Jin‐Young Park, Hye‐Min Chung, Young Woo Song, Franz‐Josef Strauss

**Affiliations:** ^1^ Department of Periodontology, Research Institute for Periodontal Regeneration, College of Dentistry Yonsei University Seoul Republic of Korea; ^2^ Innovation Research and Support Center for Dental Science Yonsei University Dental Hospital Seoul Republic of Korea; ^3^ Department of Oral Biology Medical University of Vienna Vienna Austria; ^4^ Department of Oral Medicine, Infection and Immunity Harvard School of Dental Medicine Boston Massachusetts USA; ^5^ Department of Conservative Dentistry, Faculty of Dentistry University of Chile Santiago Chile; ^6^ Clinic of Reconstructive Dentistry University of Zurich Zürich Switzerland

**Keywords:** alveolar ridge reconstruction, biomaterials, bone grafting, bone regeneration, bone substitutes

## Abstract

**Aim:**

To determine whether vertical ridge augmentation (VRA) can be obtained through guided bone regeneration (GBR) using exclusively resorbable collagen membranes and particulate bone substitutes without additional stabilization.

**Materials and Methods:**

This study retrospectively examined 22 participants who underwent VRA with staged or simultaneous implant placement. The vertical defects of all participants were filled with particulate bone substitutes and covered with resorbable collagen membranes. The augmented sites were stabilized with unfixed collagen membranes and the flap without any additional fixation. The augmented tissue height was assessed using cone‐beam computed tomography at baseline, immediately after surgery, and at annual follow‐ups.

**Results:**

The vertical bone gain of the 22 augmented sites amounted to 6.48 ± 2.19 mm (mean ± SD) immediately after surgery and 5.78 ± 1.72 mm at 1‐ to 7‐year follow‐up. Of the 22 augmented sites, 18 exhibited changes of less than 1 mm, while the other 4 showed changes of greater than 1 mm. Histological observation of three representative cases revealed new bone apposition on the remaining material.

**Conclusion:**

The present findings indicate that GBR procedures using exclusively collagen membranes and particulate biomaterials without any additional fixation are feasible options for VRA.

**What is known**

- A recent systematic review by the 15th European Workshop on Periodontology on Bone Regeneration found insufficient clinical evidence to identify the most effective technique for vertical ridge augmentation (VRA).
- Vertical ridge augmentation could be obtained using simplified clinical procedures such as guided bone regeneration with resorbable membranes. However, clinical data supporting this hypothesis are currently scarce.

**What this study adds**

- The present study provides evidence on the feasibility of VRA using exclusively collagen membranes and particulate bone substitutes without any additional fixation.

## 1 INTRODUCTION

Vertical ridge augmentation (VRA) is the most challenging intervention in implant dentistry, mostly due to its technical sensitivity and frequent complications.^1^, ^2^ Vertical ridge augmentation aims to regenerate bone volume at sites of the host chosen for implant placement, at which the bony walls are often missing. This is biologically challenging due to a lack of bony walls hindering blood clot stabilization^3^ and access to osteoprogenitor cells, which may induce inadequate bone regeneration. One attempt to overcome this biological limitation involves using autogenous bone block grafts or distraction osteogenesis.^4^, ^5^ However, these procedures are surgically invasive and are associated with increased morbidity.^1^ Therefore, simplifying the surgical procedures and reducing the invasiveness of VRA have become increasingly important. This trend is further emphasized by the shift from specialists and referral‐based clinicians to general dentists for implant dentistry.^6^ In this sense, guided bone regeneration (GBR) seems to be a logical and well‐known alternative for these complex procedures.

Guided bone regeneration is a reliable and well‐documented clinical procedure^7^, ^8^ that has been indicated as a viable alternative for VRA.^9^ Although nonresorbable membranes are considered the standard reference for GBR because of their space‐making capacity and controlled barrier function,^10^ they are often associated with soft‐tissue complications after exposure^11^, ^12^, ^13^ and require additional surgery for their removal. Consequently, resorbable membranes were proposed for VRA procedures,^9^ despite their inherent lack of a space‐making capability. To circumvent this lack of mechanical stability, resorbable membranes have been used alongside stabilizing devices (eg, pins, tenting screws, titanium mesh, or stabilizing sutures). However, these supportive devices can be difficult to install and must be removed in an additional surgery. Moreover, there is little evidence to support their use. A recent systematic review by the 15th European Workshop on Periodontology on Bone Regeneration found insufficient clinical evidence to identify the most effective technique for VRA.^14^ This means that the gold standard for VRA is yet to be determined.

Even though no technique is superior to others regarding vertical augmentation,^14^ focusing on and further exploring less‐invasive techniques for VRA such as GBR is reasonable. It can be assumed that patients tend to prefer this strategy over more‐complex procedures that cause complications and morbidity, leading to longer treatment times and higher costs. A recent relevant systematic review indicated that VRA is feasible regardless of which technique is used,^9^ and it can therefore be hypothesized that VRA can also be obtained using simplified clinical procedures such as GBR with resorbable membranes. However, clinical data supporting this hypothesis are currently scarce.

Therefore, the *aim* of the present study was to determine whether VRA can be achieved by using a simplified GBR procedure with resorbable collagen membranes and particulate bone substitutes without additional stabilization.

## 2 MATERIALS AND METHODS

### 2.1 Study design and population

Participants were eligible for inclusion if they had received either staged or simultaneous vertical augmentation procedures in either the maxillary or the mandibular region. Only participants who underwent GBR using a resorbable collagen membrane without a fixation method or device were included. Subjects were excluded if they had received GBR using autogenous/allogenous block bone grafts, titanium mesh, nonresorbable membranes, or any type of fixation such as screws or bone tacks. Participants were all treated by the same experienced oral surgeon (Jung‐Seok Lee) at the Department of Periodontology of Yonsei University Dental Hospital between 2014 and 2019. The study protocol was approved by the Institutional Review Board of Yonsei University Dental Hospital (approval no. 2‐2021‐0063), which abides by the Good Clinical Practice guidelines and the regulatory requirements. Due to the retrospective design of the study, informed consents were not necessary. The manuscript was prepared in accordance with the STROBE guidelines.

### 2.2 Surgical procedures

#### 2.2.1 Incisions

Midcrestal incisions were made on the keratinized gingiva that covered the entire vertically deficient ridge. Where gingival tissues had healed immaturely or unevenly, the incision line was displaced either buccally or palatally/lingually to include the defective soft tissue on one side of the flap to prevent it from perforating. One or two vertical‐releasing incisions were made at least one tooth away from the surgical site, and a full‐thickness mucoperiosteal flap was elevated beyond the margin of the bone defect to expose the entire defective area.

#### 2.2.2 Grafting bone substitutes and membrane coverage

**FIGURE 1:**
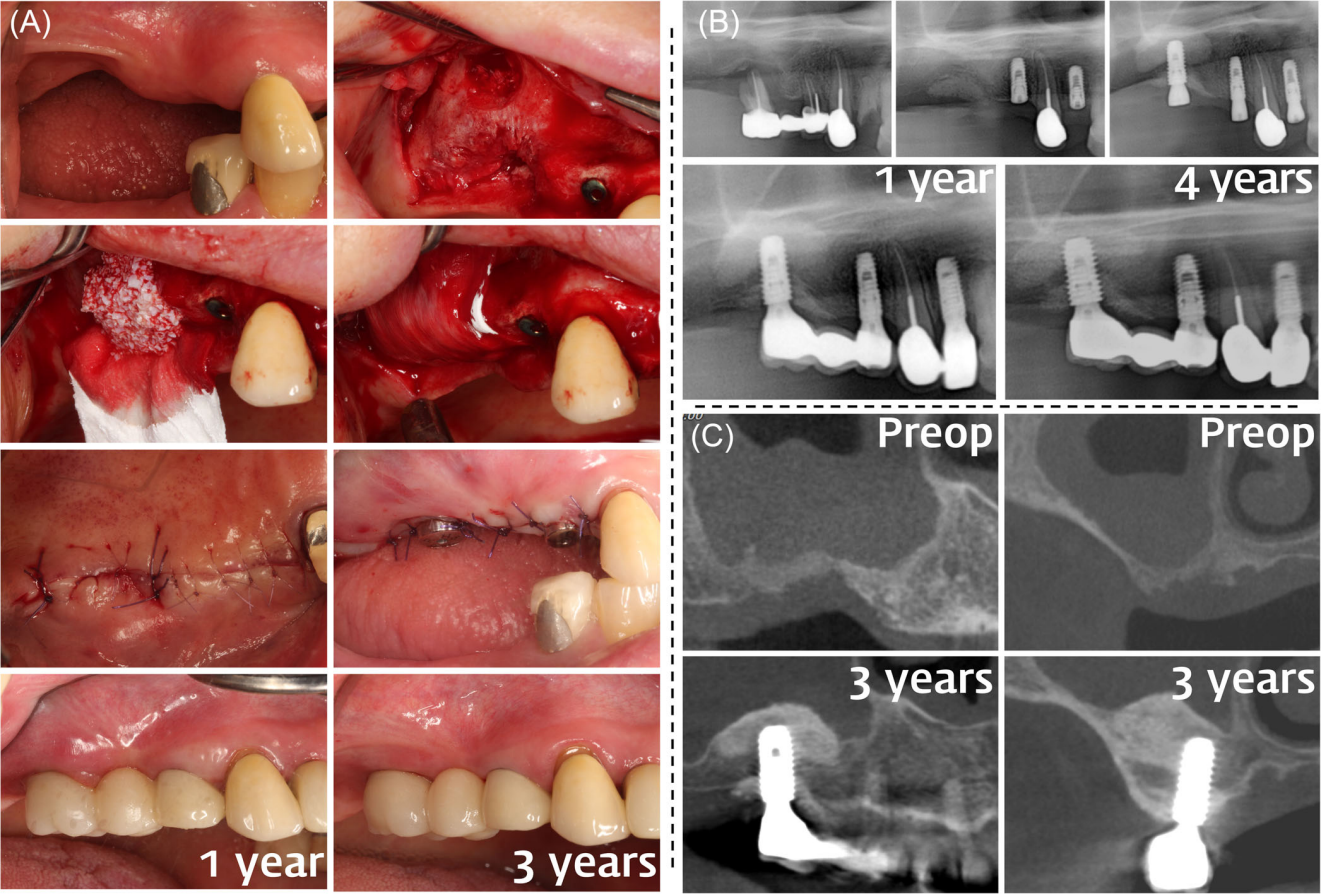
Clinical photographs (A), panoramic radiographs (B), and cone‐beam computed tomography (CBCT) imaging (C) illustrating vertical ridge augmentation (VRA) performed by guided bone regeneration (GBR) on the posterior maxilla. (A) A preoperative view indicating the vertically deficient ridges of the right posterior maxilla. A vertical bone defect was exposed after flap elevation. Sinus augmentation was performed using the lateral window technique. GBR was performed using particulate bone substitutes and resorbable collagen membranes. Membranes were placed over the bone substitute without using a fixation device. The flap was advanced using minimal releasing incisions to achieve tension‐free primary closure and was sutured. Implant fixtures were installed. A harmonious appearance was observed in the reconstructed ridges and final restorations at 1‐ and 3‐year follow‐ups. (B) Pre‐extraction radiograph indicating extensive periodontal bone loss at the upper right first molar. Radiographs of the vertically augmented sites obtained immediately postoperatively. Implant placements performed 6 months postoperatively on the upper right first molar region. Augmented peri‐implant marginal bone was well maintained at the 1‐ and 4‐year follow‐ups. (C) Preoperative cross‐sectional view of the vertical bone defects, indicating a saddle‐type morphology; coronal view of the middle of the vertical defects indicating a distinct lack of ridge height, almost level with the hard palate. A 3‐year postoperative cross‐sectional view indicating well‐maintained peri‐implant mesial/distal marginal bone levels; coronal view indicating well‐maintained buccal/palatal peri‐implant marginal bone levels

**FIGURE 2:**
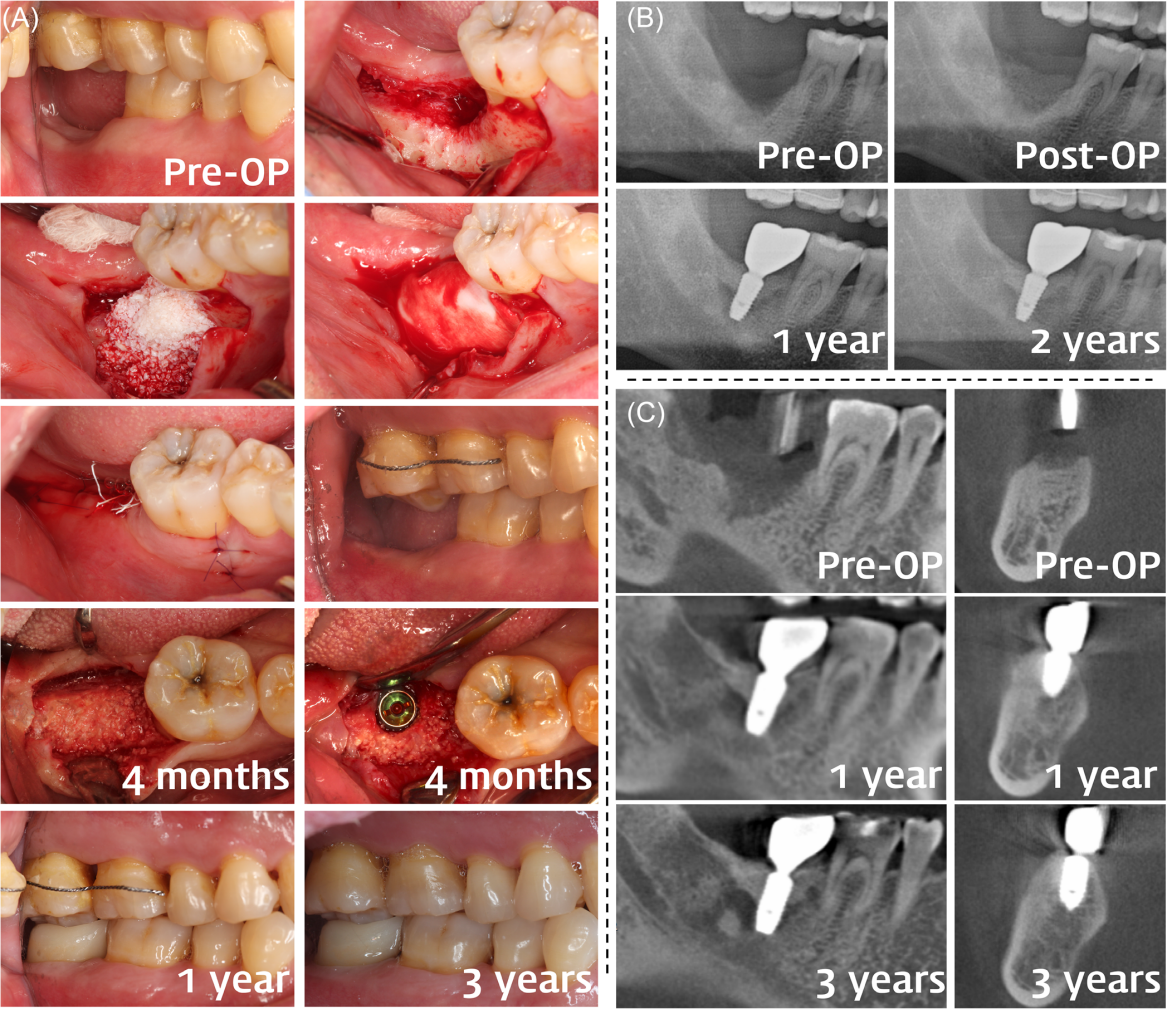
Clinical photographs (A), panoramic radiographs (B), and cone‐beam computed tomography (CBCT) imaging (C) of a staged vertical ridge augmentation (VRA) case in the posterior mandible. (A) Preoperative view of the missing lower right second molar indicating vertical ridge deficiency. After elevating the full‐thickness flap, an extensive saddle‐type bone defect was observed, including an extraction socket with delayed healing. A particulate bone substitute was grafted on the defect to restore the original height and width of the ridge. A collagen membrane was placed over the bone graft without fixation. Flap advancement was performed minimally to achieve tight primary closure and wound stability. Buccal view of the postoperative area indicating that volume of the ridge was restored. Reentry after 4 months indicating a regenerated ridge, on which staged implant placement was performed. The surgical outcomes were observed to be well maintained at 1‐ and 3‐year follow‐ups. (B) A saddle‐type ridge defect can be observed on the preoperative panoramic radiograph. Postoperative view indicating that the height of the ridge was restored. Marginal bone levels around the implant fixture were well maintained at 1‐ and 2‐year follow‐ups. (C) Sagittal and coronal cross sections of the saddle‐type ridge defect. Marginal bone levels were well maintained after 1 and 3 years

The membrane‐supporting materials used were hydrated with saline and were deproteinized bovine bone mineral (DBBM; Bio‐Oss, Geistlich, Wolhusen, Switzerland), deproteinized porcine bone mineral (DPBM; THE Graft, Purgo Biologics, Seongnam, Korea), or synthetic biphasic calcium phosphonate (BCP; Osteon II, Genoss, Suwon, Korea). The defects were filled with bone substitutes up to an extrapolated natural outline of the bony envelope extending from the outer contour to the most‐crestal point of the adjacent bone tissue attempting not to over‐augment the site. The collagen membranes were either cross‐linked collagen membrane (CCM; Collagen Membrane, Genoss) or non‐cross‐linked collagen membrane (NCCM; Bio‐Gide, Geistlich) and were trimmed to ensure a sufficient coverage of the recipient site. To ensure the grafted material was localized within the defect, the edge of the collagen membrane was tucked between the alveolar bone and the flap (Figure 1A and 2A).

#### 2.2.3 Flap‐stabilizing technique

The elevated buccal mucoperiosteal flap was advanced using periosteal‐releasing incisions at the base of the flap for passive primary closure, just enough to reach the palatal/lingual flap. By doing so, the mucoperiosteal flaps per se could provide retention and stability to the grafted materials during the initial healing period. Initially, the extent of flap advancement was assessed by placing the periosteal elevator at the height of the expected graft and pulling the flap over the instrument. If further flap mobilization was required, an additional releasing incision was made to facilitate passive primary closure.

#### 2.2.4 Sutures

Primary closure of the crestal mucoperiosteal flaps was achieved using vertical mattress sutures: 6/0 nylon (Monosyn, B. Braun, Hessen, Germany) and/or 5/0 PTFE (Biotex, Purgo Biologics). To ensure intimate flap adaptation, the near and far points of needle insertion for the vertical mattress suture were within 1 mm of the wound margin and 5 mm from the near point, respectively. Minimally advanced mucoperiosteal flaps were passively closed but supported the biomaterial grafted space without aid of fixation devices. Interrupted sutures repositioned the vertical aspects of the flap.

### 2.3 Follow‐up observations

Postoperative antibiotics (Cefaclor 250 mg) and analgesics (Ibuprofen 200 mg) were prescribed for 7 days. A clinical examination was performed 1 day after the surgery, and the sutures were removed 7–10 days later. Monthly follow‐up examinations were performed for 4–6 months until the uncovering surgery was performed. Sites that had received primary augmentation (staged GBR) received dental implants after 4–6 months. In these cases, a trephine biopsy sample was obtained whenever possible to histologically observe the new bone formed within the augmented area. Radiographic examinations were performed at annual follow‐ups by taking panoramic radiographs or cone‐beam computed tomography (CBCT) images (Figure 1B,C; Figure 2B,C). Linear measurements were made of the augmented site from the peak of the vertically augmented area to the lowest point of the preoperative alveolar ridge floor as observed in the panoramic radiograph or CBCT.

## 3 RESULTS

### 3.1 Demographic information of the included cases

This study included 22 patients, of whom 11 were males and 11 females with an age of 50.09 ± 10.02 years (mean ± SD) (43–71 years; Table 1). Of these patients, 14 were systemically healthy at the time of surgery, while 8 had a history of medical illness (3 had osteoporosis, 4 had cardiovascular diseases and were receiving anticoagulant medications, 1 had controlled type II diabetes, and 1 had chondromalacia). Vertical loss of buccal and lingual/palatal alveolar bone was present in 14 single‐tooth and 8 longer spanning edentulous sites of the included patients. Of these recipient sites, 16 were located at molars (9 and 7 in the maxilla and mandible, respectively), 4 in the maxillary premolars, and 2 in the anterior maxilla. DPBM, DBBM, and BCP were applied to 14, 7, and 1 sites, respectively. The NCCM was used in all but one site, in which CCM was used. Implants were placed with simultaneous GBR in 8 sites, and the staged approach was adopted for 14 sites. Follow‐up observation periods varied from 1 to 7 years.

**TABLE 1:** Demographic results and linear measurements in radiographs of the included patients

No.	Age/Sex	Site	PMH	Bone substitute	Membrane	Surgery type	Adverse reaction	Follow‐up period	Defect length	Defect height	Augmented height		
											After surgery	Last follow‐up	Change
1	53/F	#37	Mitral stenosis (anticoagulant medication)	DBBM	NCCM	First stage	‐	7 years 6 months	19.84 mm	3.89 mm	3.62 mm	3.58 mm	−0.04 mm
2	69/F	#17	‐	DBBM	NCCM	Second stage	Wound dehiscence	3 years	18.65 mm	5.27 mm	4.55 mm	4.42 mm	−0.13 mm
3	43/F	#16, #17	‐	DBBM	NCCM	First stage	‐	5 years	22.88 mm	4.6 mm	4.6 mm	4.68 mm	0.08 mm
4	65/M	#26	‐	DBBM	NCCM	First stage	‐	6 years	8.95 mm	5.52 mm	4.64 mm	4.65 mm	0.01 mm
5	52/M	#47	‐	DBBM	NCCM	Second stage	‐	3 years	16.5 mm	6.66 mm	7.48 mm	6.02 mm	−1.46 mm
6	59/M	#26	Hypertension (anticoagulant medication)	DPBM	NCCM	Second stage	‐	2 years 6 months	11.77 mm	9.76 mm	10.67 mm	9.92 mm	−0.75 mm
7	60/M	#47	‐	DPBM	NCCM	Second stage	‐	2 years	15.97 mm	6.98 mm	7.25 mm	6.44 mm	−0.81 mm
8	45/F	#16	‐	DPBM	NCCM	Second stage	‐	2 years	15.59 mm	5.06 mm	4.99 mm	4.35 mm	−0.64 mm
9	71/F	#14, #15	Osteoporosis (bisphosphonate medication)	DBBM	NCCM	Second stage	‐	1 year 6 months	12.23 mm	6.35 mm	5.78 mm	4.94 mm	−0.84 mm
10^a^	38/F	#27	‐	DPBM	NCCM	Second stage	‐	2 years	14.38 mm	5.38 mm	5.08 mm	5.48 mm	0.4 mm
11	69/M	#47	‐	BCP	CCM	First stage	Delayed healing	1 year 4 months	12.34 mm	7.83 mm	6.71 mm	6.64 mm	−0.07 mm
12	68/F	#16, #17	Diabetes (controlled); Osteoporosis (bisphosphonate medication)	DPBM	NCCM	First stage	Delayed healing	1 year 3 months	14.99 mm	4.94 mm	4.44 mm	4.35 mm	−0.09 mm
13	69/F	#16, #17	Osteoporosis (Bisphosphonate medication)	DPBM	NCCM	Second stage	‐	2 years	22.93 mm	7.6 mm	6.89 mm	6.06 mm	−0.83 mm
14	49/F	#36	Chondromalacia	DPBM	NCCM	Second stage	‐	2 years	12.12 mm	10.48 mm	10.53 mm	8.57 mm	−1.96 mm
15	63/M	#26, #27	‐	DPBM	NCCM	Second stage	Wound dehiscence	2 years	23.26 mm	7.51 mm	11.03 mm	6.33 mm	−4.7 mm
16	57/M	#11	Atrial fibrillation, pacemaker (anticoagulant medication)	DPBM	NCCM	First stage	‐	1 year 6 months	7.97 mm	10.22 mm	8.68 mm	8.32 mm	−0.36 mm
17	66/M	#24, #25	Brain stent (Anticoagulant medication)	DPBM	NCCM	Second stage	Delayed healing	1 year	15.81 mm	6.14 mm	5.83 mm	5.16 mm	−0.67 mm
18	55/M	#25	‐	DPBM	NCCM	Second stage	‐	2 years	10.34 mm	7.16 mm	8.02 mm	7.08 mm	−0.94 mm
19	66/M	#37	‐	DPBM	NCCM	First stage	Delayed healing	2 years	9.65 mm	4.91 mm	4.17 mm	3.94 mm	−0.23 mm
20	66/F	#13–#16	‐	DPBM	NCCM	Second stage	‐	1 year 6 months	35.37 mm	5.91 mm	4.94 mm	5.24 mm	0.3 mm
21	52/M	#21	‐	DBBM	NCCM	First stage	‐	3 years	6.51 mm	8.54 mm	7.08 mm	7.6 mm	0.52 mm
22	43/F	#35–#37	‐	DPBM	NCCM	Second stage	Delayed healing	1 year	28.69 mm	8.45 mm	5.48 mm	3.37 mm	−2.11 mm
Average ± Standard deviation									16.22 ± 7.06	6.78 ± 1.86	6.48 ± 2.19	5.78 ± 1.72	−0.70 ± 1.13

Abbreviations: BCP, biphasic calcium phosphate (Osteon II); CCM, cross‐linked collagen membrane (collagen membrane); DBBM, deproteinized bovine bone mineral (Bio‐Oss); DPBM, deproteinized porcine bone mineral (THE Graft); NCCM, non‐crosslinked collagen membrane (Bio‐Gide); PMH, past medical history.

^a^ One case showed early implant failure (3 months) of a transmucosally placed implant, but the replaced implant is in a clinically successful state thereafter.

### 3.2 Clinical and radiographic observations

**FIGURE 3:**
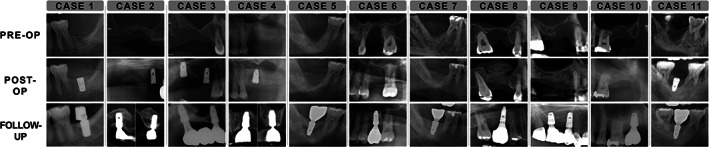
Radiographic findings for cases 1–11, including their preoperative, postoperative, and latest follow‐up data

**FIGURE 4:**
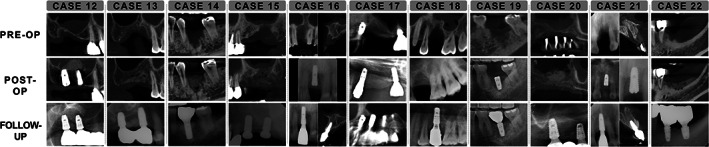
Radiographic findings for cases 12–22, including their preoperative, postoperative, and latest follow‐up data

Table 1 lists the detailed information of all sites. The preoperative, postoperative, and follow‐up radiographs of all cases are shown in Figures 3 and 4. All cases indicated successful vertical augmentation of the alveolar ridge; the augmented height was 6.48 ± 2.19 mm immediately after surgery, which was well maintained during follow‐ups up to 7 years (5.78 ± 1.72 mm). Most sites indicated limited changes (<1 mm) in vertical ridge height, with the exception of four (cases 5, 14, 15, and 22). Of these four cases, three (cases 5, 14, and 15) had apparent over‐augmentation, with grafted bone substitutes outside the bony envelope defined by the level of attachment to the adjacent teeth. Thereafter, regenerated bone was formed at the level of the bony attachment, and substantial shrinkages were observed in these sites. At the remaining site (case 22), a large ulcerative lesion occurred at the margins of both flaps resulting in delayed healing.

Among the 22 cases, 15 sites healed uneventfully, but the other 7 exhibited soft‐tissue complications during the initial healing period. Two maxillary molar sites (cases 2 and 15) presented small wound dehiscence (2–3 mm) but healed completely thereafter (cases 2 and 15). Delayed healing, manifested as sloughing and granulation tissue formation in the area surrounding the sutured flap margins, occurred at five other sites [three mandibular molar sites (cases 11, 12, and 17), one maxillary molar (case 19), and one maxillary premolar (case 22)]. One of the above‐mentioned sites presented an ulcerative lesion (case 22), while the other four compromised sites presented a well‐maintained augmented alveolar ridge with less than 1 mm of change (−0.07, −0.09, −0.81, and −0.23 mm for cases 11, 12, 7, and 19, respectively) in radiographic height up to 3 years after the implant surgery.

Failure in osseointegration caused one dental implant to be removed 3 months after the implant surgery, in which a bone level implant (5.0‐mm diameter and 8.5‐mm length; Luna; Shinhung Implant System, Seoul, Korea) was placed with healing abutment connection and simultaneous crestal sinus floor elevation at 6 months after VRA (case 10). After 5 months of healing, another bone level implant (5.0‐mm diameter and 8‐mm length; Superline; Dentium, Seoul, Korea) was placed. Despite the removal and replacement of the dental implant, the vertically augmented alveolar ridge was maintained without any crestal bone loss. In general, all implants revealed successful clinical results with virtually no marginal bone loss around the implant at 1–7.5 years, including the replaced dental implant in case 10.

### 3.3 Histological observations

**FIGURE 5:**
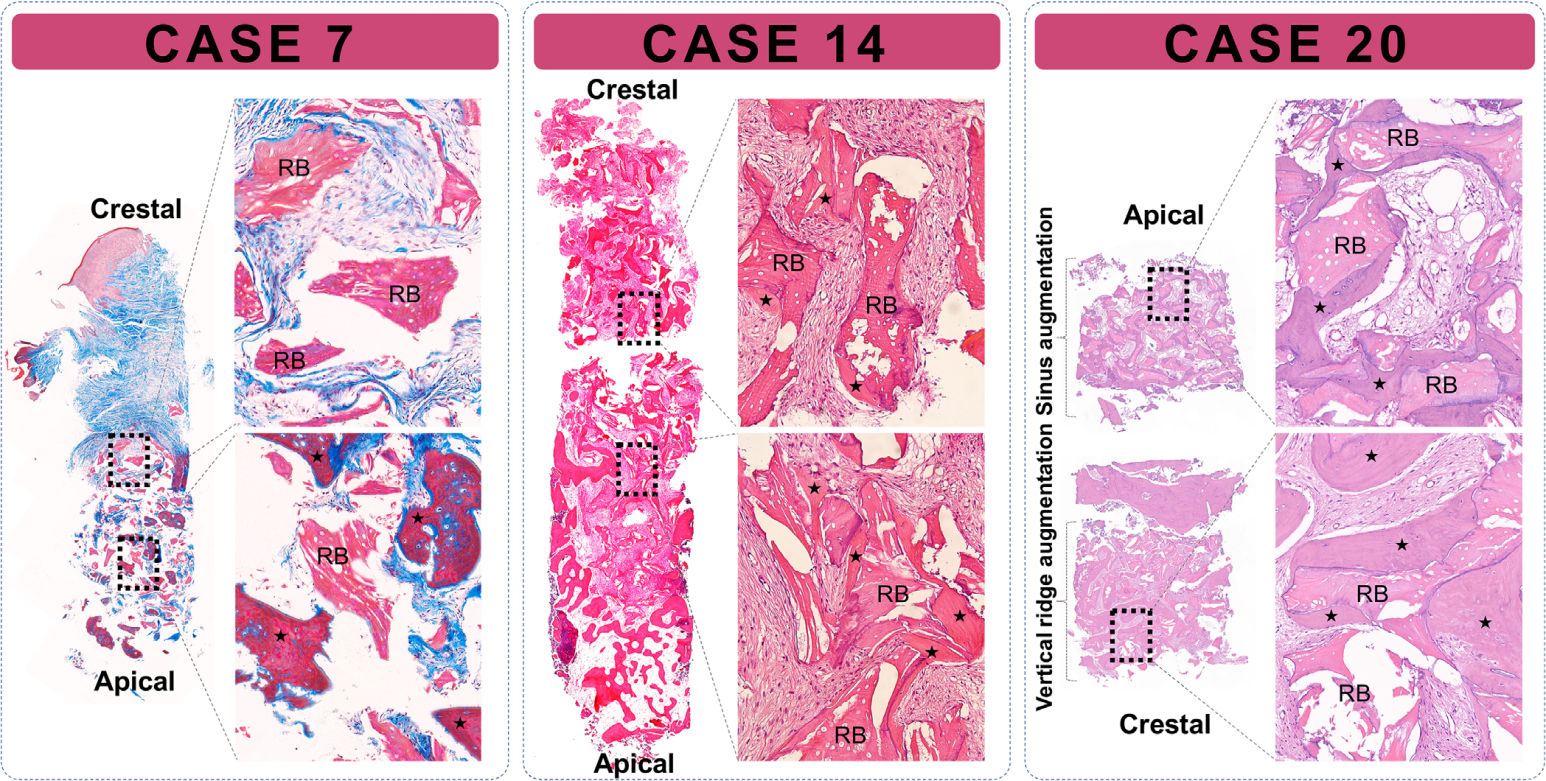
Histological view of the trephine biopsy samples obtained from three sites grafted using deproteinized porcine bone mineral (cases 7, 14, and 20). Substantial new bone formation surrounding the residual biomaterials was observed in all of these cases. New bone appeared to expand from the basal bone; at 4 months postoperatively, the residual biomaterials in the crestal region were surrounded by dense connective tissue. In case 20, the trephine biopsy sample included both the vertically augmented ridge and augmented sinus. The main difference between the two sites was in the consistency of the connective tissue, which was denser at the vertically augmented site. Asterisk represents newly regenerated bone; case 7, Masson's trichrome staining; cases 14 and 20, hematoxylin and eosin staining; RBs, residual biomaterials

Histological biopsy samples were obtained using trephines during implant preparation for three sites of the 14 staged‐approach cases that had been augmented using DPBM. Two samples were obtained from augmented single mandibular molar sites (cases 7 and 14). These histological examinations indicated that substantial new bone formation occurred around the residual biomaterials (Figure 5). Greater amounts of newly formed bone were observed in the deeper areas proximal to the original alveolar bone (lower boxes in cases 7 and 14), whereas minimal new bone formation was observed at the coronal area; only the residual biomaterials were present at the most‐coronal area of both the mandibular biopsy samples (upper boxes in cases 7 and 14). Two biopsy samples were collected in another case with multiple missing posterior maxilla teeth (case 20): from a VRA and a sinus augmentation site. Both sites exhibited substantial new bone formation around the residual biomaterial; however, there was a difference in the appearance of the connective tissues surrounding the augmented bone (Figure 5). While the biopsy sample from the augmented sinus area presented loose connective tissue with abundant vessels (upper box), the GBR site sample was filled with dense connective tissues (lower box) similar to the sites from cases 7 and 14.

## 4 DISCUSSION

This study retrospectively assessed the clinical, radiographic, and histological records of a single cohort of 22 patients who had received simplified VRA by GBR using conventional collagen membranes and particulate bone substitutes without additional fixation (eg, pins, titanium mesh, or tenting screws). This study found that (1) VRA is feasible when applying GBR using exclusively resorbable membranes and particulate bone substitutes without any additional fixation, (2) VRA outcomes are favorable and stable for up to 7 years, and (3) histologically, appositional new bone growth was observed around the bone substitute particles.

Despite the lack of additional fixation devices (eg, pins or titanium mesh) in the present study, stabilizing the graft materials remains a key factor in bone regeneration. In all of the treated cases, the mucoperiosteal flaps were minimally but tightly advanced to stabilize the grafting materials during the healing period. Conversely, exaggerated flap advancement inducing slackness may have altered the new bone formation due to masticatory forces and swelling displacing the grafting material. Traditionally, resorbable pins, bone tacks,^15^, ^16^ and membrane‐stabilizing mattress sutures^17^ have been recommended for stabilizing the collagen membrane. However, the grafted biomaterials can also be stabilized using the mucoperiosteal flap and residual ridge.^15^ For example, in both short and long saddle‐type defects, which somewhat mimic vertical ridge defects, good membrane stabilization can lead to successful GBR even without the use of a bone substitute material, as demonstrated by the hallmark study of Schenk and coworkers.^18^

The morphology and size of the vertical bone defects in the present study differed markedly. In this sense, the regenerative potential and the stability of the grafted site might be predicted by the availability of bony peaks surrounding the defect as these peaks provide additional mechanical stability. Moreover, one‐third of the augmented sites were bounded by teeth having bone attachments at the coronal level. These anatomical aspects may have facilitated the regeneration and contributed to the mean vertical gain of about 5.7 mm, which was greater than the mean vertical gain of about 4 mm found in previous systematic studies that employed GBR.^9^, ^14^ This indicates that morphology is linked to the regenerative potential. A recent clinical study analyzed biopsy samples obtained from damaged sockets at 4 months after bone grafting and found a positive correlation between residual height and the amount of newly formed bone.^19^ Those authors concluded that residual ridge morphology played a critical role in the regenerative potential of the augmented sites.

The crucial hurdles for vertical augmentation are the serious intra‐ or postoperative complications such as graft material exposure or infection, which result in the need for regrafting. The generally recommended techniques of distraction osteogenesis or autogenous block bone graft have been associated with a high risk of serious complications.^14^ Guided bone regeneration is known to produce fewer complications, but failures or minimal bone gain should be expected when these complications involve nonresorbable membranes.^20^ Although the present cohort presented a relatively high rate of complications (32%), these were not considered serious^14^ and thus did not affect the clinical outcomes. These complications included sloughing without grafted material exposure and 2–3 mm wound dehiscence, which completely healed within 4 weeks. Using a collagen membrane (NCCM in all but one case) may have reduced the number of complications,^12^ resulting in a substantial vertical ridge gain (4.89 ± 1.22 mm).

Over‐augmentation of the vertical defect was prone to cause greater dimensional shrinkage; all three cases presenting grafted materials beyond the extrapolated line between the most‐coronal points of the adjacent alveolar ridge (bony envelope) demonstrated reductions of vertical ridge height exceeding 1 mm (cases 5, 14, and 15). In the radiographs from the final visits, the augmented ridges were maintained at the same level as the extrapolated line of the bony envelope regardless of the presence of over‐augmentation in the materials. This is consistent with a recent autopsy study that histologically demonstrated new bone formation mostly occurring within bony envelopes despite the retention of over‐augmented biomaterials over several years.^21^

The gain values obtained in the present study were lower than those found in other techniques used for VRA, such as distraction osteogenesis and bone blocks, which was consistent with previous reports. A systematic review with meta‐analysis compared these two techniques with GBR and indicated that distraction osteogenesis produced the greatest bone gain (8.04 mm) but also the highest complication rate (47.3%).^14^ In contrast, the same review revealed that GBR had the lowest complication rate (12.1%) and a substantial bone gain (4.18 mm). Although distraction osteogenesis shows the greatest bone gains, patients tend to prefer alternative treatments that have fewer complications, are less invasive, and avoid a donor‐site surgery.^22^ The reported bone gains using GBR are much smaller than those of distraction osteogenesis. Nevertheless, the clinician should not necessarily select the treatment option with greatest efficacy but rather the option with less morbidity in accordance with the patient's preferences.^23^ Furthermore, due to the familiarity with the technique, GBR has become the most favored treatment choice by not only the referral‐based clinicians but also the specialists. In this context, and based on the present findings, GBR utilizing the flap‐stabilizing technique might be a viable method for VRA.

This study has some limitations that should be considered when interpreting the present findings. Firstly, it has a retrospective design, with a single operator and 1 cohort of 22 patients. Also, three different types of bone substitutes were used, which might interfere with the interpretations of the present results. It should be noted, however, that previous studies have shown comparable efficacy of these three bone substitutes.^19^, ^24^ Moreover, the mean difference (−0.7 ± 1.13 mm) between the different substitutes was minimal, indicating a similar regenerative capacity. Secondly, biopsy samples were not available for all cases, and bone regeneration could therefore not be confirmed despite the favorable radiographic results. Thirdly, most of the cases were still midway through their follow‐up period. Finally, it is difficult to standardize the flap advancement method for the stabilization of the grafted materials. Therefore, more studies are needed with a prospective controlled trial design including multiple institutions and longer observation periods to further evaluate the exclusive use of collagen membranes and particulate bone substitutes as clinical modalities for VRA.

In the present 22 patient cohort, the exclusive use of collagen membrane and particulate bone substitutes resulted in ≈5 mm of VRA with a low complication rate. The histological bone formation and observed ridge stability in the present study indicates the feasibility of VRA by using exclusively collagen membranes and particulate bone substitutes.

## CONFLICT OF INTEREST

The authors declare no conflicts of interests.

## AUTHOR CONTRIBUTIONS

Jung‐Seok Lee conceived the ideas; Hae‐Min Jung and Young Woo Song collected the data; Hae‐Min Jung and Jin‐Young Park analyzed the data; and Jung‐Seok Lee and Jin‐Young Park led the writing; Franz‐Josef Strauss reviewed and edited the manuscript.

## Data Availability

The data that support the findings of this study are available on request from the corresponding author. The data are not publicly available due to privacy or ethical restrictions.
